# Temporal Dynamics of Abiotic and Biotic Factors on Leaf Litter of Three Plant Species in Relation to Decomposition Rate along a Subalpine Elevation Gradient

**DOI:** 10.1371/journal.pone.0062073

**Published:** 2013-04-19

**Authors:** Jianxiao Zhu, Wanqin Yang, Xinhua He

**Affiliations:** 1 Insititute of Ecological Forestry, Sichuan Agricultural University, Chengdu, China; 2 Key Laboratory for Earth Surface Processes, Ministry of Education, Department of Ecology, Peking University, Beijing, China; 3 School of Life Sciences, Yunnan Normal University, Kunming, Yunnan, China; 4 School of Plant Biology, University of Western Australia, Crawley, WA, Australia; DOE Pacific Northwest National Laboratory, United States of America

## Abstract

Relationships between abiotic (soil temperature and number of freeze-thaw cycles) or biotic factors (chemical elements, microbial biomass, extracellular enzymes, and decomposer communities in litter) and litter decomposition rates were investigated over two years in subalpine forests close to the Qinghai-Tibet Plateau in China. Litterbags with senescent birch, fir, and spruce leaves were placed on the forest floor at 2,704 m, 3,023 m, 3,298 m, and 3,582 m elevation. Results showed that the decomposition rate positively correlated with soil mean temperature during the plant growing season, and with the number of soil freeze-thaw cycles during the winter. Concentrations of soluble nitrogen (N), phosphorus (P) and potassium (K) had positive effects but C:N and lignin:N ratios had negative effects on the decomposition rate (*k*), especially during the winter. Meanwhile, microbial biomass carbon (MBC), N (MBN), and P (MBP) were positively correlated with *k* values during the first growing season. These biotic factors accounted for 60.0% and 56.4% of the variation in decomposition rate during the winter and the growing season in the first year, respectively. Specifically, litter chemistry (C, N, P, K, lignin, C:N and lignin:N ratio) independently explained 29.6% and 13.3%, and the microbe-related factors (MBC, MBN, MBP, bacterial and fungal biomass, sucrase and ACP activity) explained 22.9% and 34.9% during the first winter and the first growing season, respectively. We conclude that frequent freeze-thaw cycles and litter chemical properties determine the winter decomposition while microbe-related factors play more important roles in determining decomposition in the subsequent growing season.

## Introduction

Litter decomposition is a fundamental biogeochemical process and plays an important role in the terrestrial carbon (C) cycle [1]–[3]. The ongoing global warming will increase the decomposition rate of litter, particularly under cold biomes such as high latitude and altitude alpine forests, which accumulate large amounts of dead plant material and soil organic C [4]. An increase of associated CO_2_ release into atmosphere with an increased litter decomposition rate could have profound repercussions for the subalpine and alpine forest [5], [6]. Studies have revealed that the initial litter quality, including C and nitrogen (N) concentrations [7], C:N ratio [8], lignin concentrations [9] and lignin:N ratio [1], [10], regulates the litter decomposition process. Meanwhile, a greater decomposable litter with high N but low C and lignin would support greater microbial biomass and decomposer communities, which in turn would enhance litter decomposability. In addition, the subsequent quality of litter that is produced during the decomposition and senescence process has also been studied in a given biome [11], [12]. Moreover, litter decomposition process and rate may change with the change of soil temperature, which directly affects on microbial communities and litter chemistry [13]. To better understand whether the decomposing litter quality and microbial compositions are important drivers of decomposition in the subalpine forests, a test needs to be explored: whether the changed litter quality and the accompanied changed composition of the litter microbial communities in litter correlate with the decomposition rate among species and elevations.

Environmental gradients, especially elevation gradients, have helped to qualify the influence of environmental conditions on forest ecosystem processes [14]–[17]. Along a 2,800 m elevation gradient in Peruvian tropical forests with no constraints on soil moisture and little seasonality in soil temperature, temperature plays the most important role in leaf litter decomposition [18]. However, theoretically, the severely low temperature seriously limits the processes of material cycling, including litter decomposition, particularly during winter and early spring in subalpine and alpine regions [19]. In contrast, physical and chemical losses of organic compounds from leaching and other processes related to the duration of freezing and thawing in winter may change litter quality and contribute to litter decomposition [20]–[22]. As a consequence, the drivers of litter decomposition during the subsequent growing season following a freezing and thawing stage may be different among elevations. Unfortunately, less attention has been paid to the ecological linkages of litter decomposition between winter and a subsequent growing season [23], [24].

On the other hand, soil freezing and thawing event during winter is one of the most significantly environmental factors on litter decomposition in subalpine regions [22], [25]. Repeated soil freeze-thaw cycles (FTCs) that destroy microbial cells would release nutrients for the surviving microbes, which are highly active during the thawing stage [26]. In general, changed litter microbial biomass and decomposers play more central roles in determining a later litter decomposition during the growing season [13]. However, information about the effects of elevation variations of soil FTCs and the changed litter quality on the subsequent decomposition is limited.

In this study, we focus on the effects of changing litter chemistry, microbial biomass, extracellular enzymes and decomposer communities on the decomposition rate at sampling dates. We also focus on the effects of soil freeze-thaw cycles during the winter and the altered litter quality during the subsequent growing season on litter decomposition. To examine whether changes in litter quality over time affect litter decomposability, in a field decomposition experiment using senescent leaf litter from native spruce (*Picea asperata*), fir (*Abies faxoniana*) and birch (*Betula albosinensis*), we studied the temporal changes in litter chemistry and microbial composition in decomposing litter along an elevation gradient in subalpine and alpine forests in the Bipenggou Nature Reserve (E102°53′–102°57′, N31°14′–31°19′, 2458–4619 m a.s.l.), Sichuan, China. Here, we hypothesized that 1) soil temperature and FTCs could increase the litter decomposition rate during winter, and change the litter chemistry and litter microbial composition after winter; and 2) the drivers of litter decomposition differ between winter and the subsequent growing season in subalpine forests.

## Materials and Methods

### Ethics Statement

The Institute of Ecological Forestry, Sichuan Agricultural University has had a permit from the Western Sichuan Forestry Bureau to conduct scientific experiments in the Bipenggou Nature Reserve since March 2006. The senescent fresh leaf litter collected for this study was only sampled at a very limited scale, and thus, had negligible effects on broader ecosystem functioning. Moreover, this research was carried out in compliance with the laws of People’s Republic of China. The research did not involve measurements on humans or animals and no endangered or protected plant species was involved.

### Site Description

This study site is located in the Bipenggou Nature Reserve, a transitional area between the Qinghai-Tibet Plateau and the Sichuan Basin, southwest China. The mean annual temperature is 3°C with maximum and minimum temperatures of 23°C (July) and –18°C (January), respectively. Annual precipitation is about 850 mm. The cold season starts in November as temperature drops below 0°C after snow falls and the soil remains frozen for a period of 5 to 6 months. The basic vegetation and soil information is listed in the Table 1.

**Table 1:** Basic geography, vegetation and soil along the four subalpine and alpine forests in the Bipenggou Nature Reserve, Sichuan, China.

	Elevation (m)	Aspect	Slope	Vegetation	Soil
Site 1	3582	NE45°	34°	Tree canopy dominated by *Abies faxoniana*(∼120 years age) with dominated understory*Rhododendron delavayi* and *Berberis* spp.	Cambisols with A–C layers, 15±2 cm organic layer, pH 6.2
Site 2	3298	NE42°	31°		
Site 3	3023	NE38°	24°	Tree canopy dominated by *Abies faxoniana* (∼70 years age) with dominated understory *Fargesia spathacea*	Cambisols with A–C layers, 12±1 cm organic layer, pH 6.5
Site 4	2704	NE36°	35°	Tree canopy dominated by *Betula albosinensis* and*Picea asperata* (∼60 years age) with dominatedunderstory *Fargesia nitida*	Cambisols with oA–C layers, 12±1 cm organic layer, pH 6.3

### Leaf Litter Decomposition Experiment

The litterbag technique was used to quantify the leaf litter decomposition rate in four selected subalpine forests with similar canopy densities [27]. These four forests are distributed over a 900 m vertical transition zone at about 2,700 m, 3,000 m, 3,300 m and 3,600 m elevation (Table 1). In October 2008, fresh senescent leaves of spruce, fir and birch were collected from the floor of the selected four forests. After two-weeks the air-dried litter was placed inside 0.50 mm nylon mesh bags (20×20 cm, 15 g for spruce and fir or 10 g for birch per bag) and then the bags were sealed. We explored 5 plots (2×2 m^2^) from the four forests to place the litter bags. A total of 1,020 litter bags (4 elevations ×3 species ×17 sampling date ×5 replicates) were placed on the floor of the four forests on 6 November 2008. Dry weight of litter was determined by oven-drying (70°C, 48 h), and litter moisture values were 9.51±0.00%, 9.15±0.01% and 9.05±0.01% for spruce, fir and birch, respectively. Litter bags were collected after 32, 138, 167, 195, 234, 275, 306, 343, 371, 402, 441, 490, 513, 538, 563, 648 and 740 days of placement for the decomposition determination.

**Figure 1:**
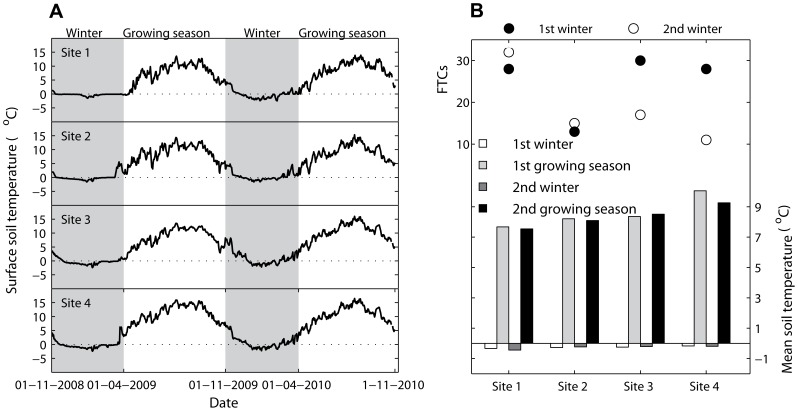
Variations of surface soil temperature (A), mean seasonal soil temperature (columns) and numbers of soil freeze-thaw cycles (FTCs) (dots) (B) during each stage of decomposition at the four study sites (elevations).

Soil temperature close to the litterbags was measured every 2 hours from 6 November 2008 to 12 November 2010 (Figure 1A) using an iButton DS1923-F5 Recorder (Maxim Integrated Products, Inc., San Gabriel Drive Sunnyvale, USA) that was placed on the forest floor. A freeze-thaw cycle was defined whenever the temperature dropped below 0°C for at least 3 h and followed by a rise above 0°C for at least 3 h, and vice versa (Figure 1B) [28].

### Chemical Analysis

Samples were stored at 4°C and analyses were finished within 2 weeks. Foreign materials such as the ingrown roots, soils debris and microbial hyphae in the litter bags were carefully removed. A quartering method was used to take out residual litter from the remaining biomass for relevant measurements. The sampled litter was oven-dried at 70°C for 48 hours to a constant weight, and then ground (1 mm sieve) for C, N, P, K, and lignin analysis [29].

### Analysis of Microbial Biomass, Extracellular Enzymes and Decomposer Communities

Determination of the numbers of colony forming units (CFUs) of fungi and bacteria in fresh sampled litter were according to the plate culture method [30]. Microbial biomass C (MBC), microbial biomass N (MBN) and microbial biomass P (MBP) in fresh sampled litter were extracted by the chloroform fumigation-extraction and then analyzed by the indigotic colorimetry method [31], [32]. The correction factors were *K*_C_ = 0.30 for MBC, *K*_N_ = 0.45 for MBN and *K*_P_ = 0.40 for MBP, respectively.

Determination of activity of sucrase (mg g^−1^ h^−1^) and acid phosphatase (ACP, pH 6.5, mg g^−1^ h^−1^) was according to Guan [33] with modifications. Briefly, 1.0 g litter was incubated at 37°C for 24 h with 15 ml 8% sucrose and acetate buffer for sucrase or 25 ml 0.5% disodium phenyl phosphate buffer for ACP.

### Calculations of Decomposition Rate

In order to compare decomposition rates among plant species and elevations in relation to decomposing litter quality, the decomposition rate (*k*) for each litter was determined during each decomposition stage [34]:
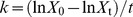
where *X*_0_ is the biomass amount of the original litter, *X*_t_ is the biomass remaining at time *t*, and *t* is the period between the two measurements at each stage (in years). The *k* values were calculated for each stage (the first or second winter, the first or second growing season) for the individual litter species at each elevation site.

### Statistical Analysis

At each decomposition stage (the first or second winter, the first or second growing season) the effects of elevation and species on the decomposition rate *(k*) were analyzed using a two-way ANOVA model. To explore the effects of elevation gradient on the factors in the three litter types with relation to litter decomposition during each decomposition stage, the Pearson’s correlation coefficients were calculated between elevations and biotic (C, N, P, K, lignin MBC, MBN, MBP, bacterial and fungal biomass, sucrase and ACP activity) or abiotic factors (mean soil temperature and FTCs). We also focused on model explanatory power (proportion of deviance explained) using a partial regression method [35]. Three separate multiple regression analyses were applied to assess the relative explanatory power of litter chemistry and microbe-related factors on the decomposition rate (*k*). All of these had the same response variable, but each analysis used a different set of the explanatory variables, including (I) the litter chemistry variables (C, N, P, K and lignin in the decomposing litter) only, (II) the microbe-related variables (microbial biomass, extracellular enzymes and decomposer compositions in the decomposing litter) only, and (III) all of these explanatory variables used in (I) or (II). Comparing the R^2^ values from these three analyses allowed us to partition the variance of the response variables to four fractions. Fraction a is explained uniquely by the litter chemistry and equals R^2^(III) – R^2^(II). Fraction b is explained jointly by the litter chemistry and the microbe-related factors and equals to R^2^(I)+R^2^(II) – R^2^(III). Fraction c is explained uniquely by the microbe-related factors and equals to R^2^(III) – R^2^(I). Fraction d is unexplained by the available biotic factors (litter chemistry and microbe-related factors) and equals to 100% – R^2^(III). All these analyses of variances and regressions were performed in Matlab R2012a (The MathWork Inc., Natick, MA).

## Results

### Decomposition rate of Litter in Relation to Soil Surface Temperature and Soil Freeze-thaw Cycles

**Figure 2:**
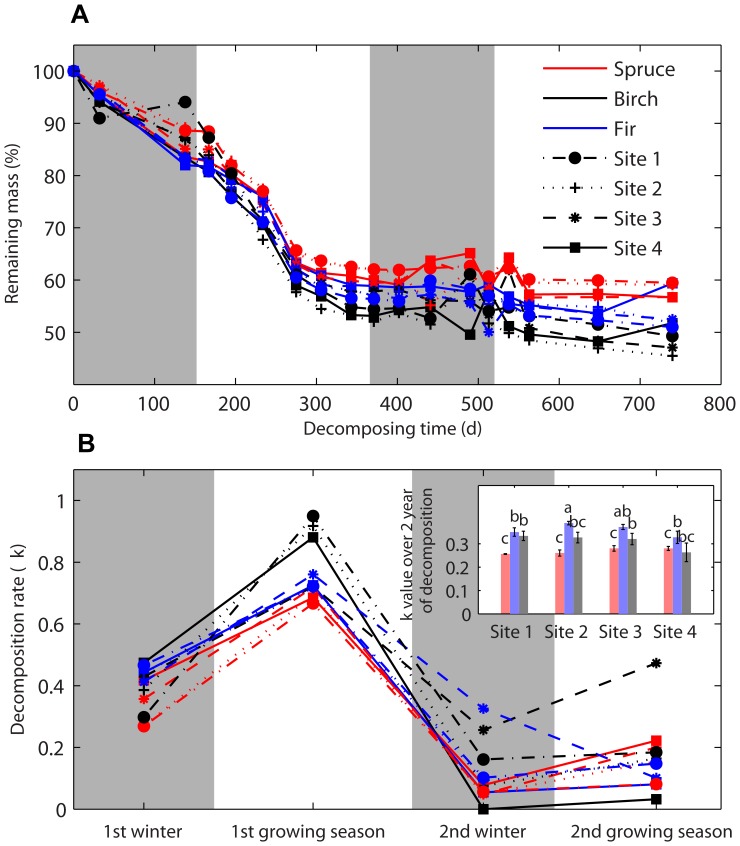
Percentage of biomass remaining of leaf litter (A) and the decomposition rate (*k*) during each stage of decomposition at the four elevations (B). Insert figure is the *k* values of the 2 year of decomposition (means ± SE, n = 5) and different letters denote significant differences at *P*<0.05.

Over the 2-year decomposition, the variation of the decomposition rate (*k*), averaged across all litter types at each elevation site, is shown in Figure 2. The mean values of *k* ranged from 0.26 to 0.40, with significant differences among species but not among elevations (*F*_2, 48_ = 20.895, *P*<0.001 and *F*_2, 48_ = 2.007, *P* = 0.125 for species and elevations respectively, two-way ANOVA, Table 2). However, statistical differences between elevation and *k-*values were detected from decompositions during each stage (except for the first growing season, two-way ANOVA, Table 2). The highest *k*-values (0.67–0.88) occurred during the first growing season, when soil temperature was high (Figure 1). However, there was also a relatively high decomposition rate (0.23–0.60) during the winter with frequent freezing and thawing cycles (Figure 1, 2).

**Table 2:** Results of the two-way ANOVA for the effects of elevation and species treatments and their interactions on the leaf litter decomposition rate (*k*).

Sources of deviation	1st winter			1st growing season			2nd winter			2nd growing season			Total 2 years		
	df	*F*	*P*	df	*F*	*P*	df	*F*	*P*	df	*F*	*P*	df	*F*	*P*
Elevation	3	2.881	**0.012**	3	0.819	0.49	3	5.204	**0.003**	3	5.943	**0.029**	3	2.007	0.125
Species	2	4.812	**0.045**	2	21.416	**<0.001**	2	0.965	0.388	2	3.808	**0.002**	2	20.895	**<0.001**
Interaction	6	1.045	0.408	6	3.088	**0.012**	6	2.11	0.069	6	4.25	**0.002**	6	1.43	0.223

Bold *P*-values indicate significant effects (*P*<0.05).

Elevation exerted significantly negative effects on the mean soil temperature during each stage (*r* values ranged from −0.99 to −0.86, *P*<0.05, Table 3), but had different effects on soil FTCs between the two winters of decomposition (*r*_1st winter_ = −0.28 and *r*_2nd winter_ = 0.64, *P*<0.05, Table 3). Numbers of soil freeze-thaw cycles (FTCs) at the four elevations displayed positive effects on *k* values during both winters (*r*_1st winter_ = 0.34, *r*_2nd winter_ = 0.28, *P*<0.05, Table 4). However, *k* values were not affected by soil mean temperature during the two winter seasons. In contrast, soil mean temperature was significantly positively correlated with *k* values during the two growing seasons (*r*_1st winter_ = 0.49, *r*_2nd winter_ = 0.38, *P*<0.05, Table 4).

**Table 3:** Pearson’s correlation coefficients (*r*) between elevation and biotic or abiotic factors in decomposing litter.

	1st winter	1st growing season	2nd winter	2nd growing season
C	0.06	−0.16*	−0.33*	−0.28*
N	−0.08	−0.32*	0.12*	0.11
P	−0.05	0.00	0.00	0.03
K	0.03	0.22*	0.21*	0.15*
Lignin	−0.24*	−0.02	−0.01	0.03
C:N	0.11	0.26*	−0.17*	−0.16*
Lignin:N	0.01	0.28*	−0.06	−0.05
MBC	0.40*	0.15*	−0.16*	−0.13*
MBN	0.08	0.09	−0.08	0.01
MBP	0.11	−0.09	0.07	0.07
Bacteria	−0.14	−0.15*	0.16*	0.33*
Fungi	−0.17*	0.26*	−0.08	0.23*
Sucrase activity	0.09	−0.02	0.01	0.02
ACPA	0.10	0.23*	−0.13*	0.15*
Soil temperature	−0.98*	−0.91*	−0.86*	−0.99*
FTCs	−0.28*	N.A.	0.34*	N.A.

An asterisk (*) indicates significant difference at *P*<0.05.

Abbreviations: FTCs = numbers of soil freeze-thaw cycles, MBC = microbial biomass carbon, MBN = microbial biomass nitrogen, MBP = microbial biomass phosphorus, and ACPA = acid (pH 6.5) phosphatase (ACP) activity. An asterisk (*) indicates statistically significant (*P*<0.05).

**Table 4:** Pearson’s correlation coefficients (*r*) between biotic or abiotic factors and decomposition rate (*k*).

	1st winter	1st growing season	2nd winter	2nd growing season
C	−0.01	−0.07	−0.17	−0.17
N	0.54*	0.26*	0.36*	−0.20
P	0.37*	0.32*	−0.10	0.17
K	0.17	0.39*	−0.03	−0.08
Lignin	−0.13	−0.27*	−0.07	−0.21
C:N	−0.55*	−0.35*	−0.21	0.13
Lignin:N	−0.50*	−0.40*	−0.31*	0.15
MBC	0.32*	0.70*	0.26*	0.13
MBN	0.02	0.29*	0.02	0.21
MBP	0.27*	0.32*	0.10	0.06
Bacteria	0.17	0.13	−0.24	0.35*
Fungi	0.26*	0.33*	−0.11	0.37*
Sucrase activity	0.09	0.44*	0.03	0.11
ACPA	0.13	0.68*	−0.07	0.09
Temperature	0.21	0.49*	−0.16	0.38*
FTCs	0.34*	N.A.	0.28*	N.A.

An asterisk (*) indicates significant difference at *P*<0.05.

Abbreviations: FTCs = numbers of soil freeze-thaw cycles, MBC = microbial biomass carbon, MBN = microbial biomass nitrogen, MBP = microbial biomass phosphorus, and ACPA = acid (pH 6.5) phosphatase (ACP) activity.

### Decomposition Rate of Litter in Relation to Biotic Factors

The Pearson’s correlation coefficients (*r*) between elevation and biotic factors are shown in Table 3. Elevation exerted a significantly negative effect on C but positive effect on K concentration in the litter after the first winter of decomposition (*r* = −0.16 to −0.33). Moreover, the *r* values between elevation and C:N or lignin:N ratio were 0.26 and 0.28 during the first growing season of decomposition, respectively. The relationship between elevation and MBC was significantly positive during the first year (*r* = 0.15 to 0.40), but negative (*r* = −0.13 to −0.40) during the second year of decomposition.

**Figure 3:**
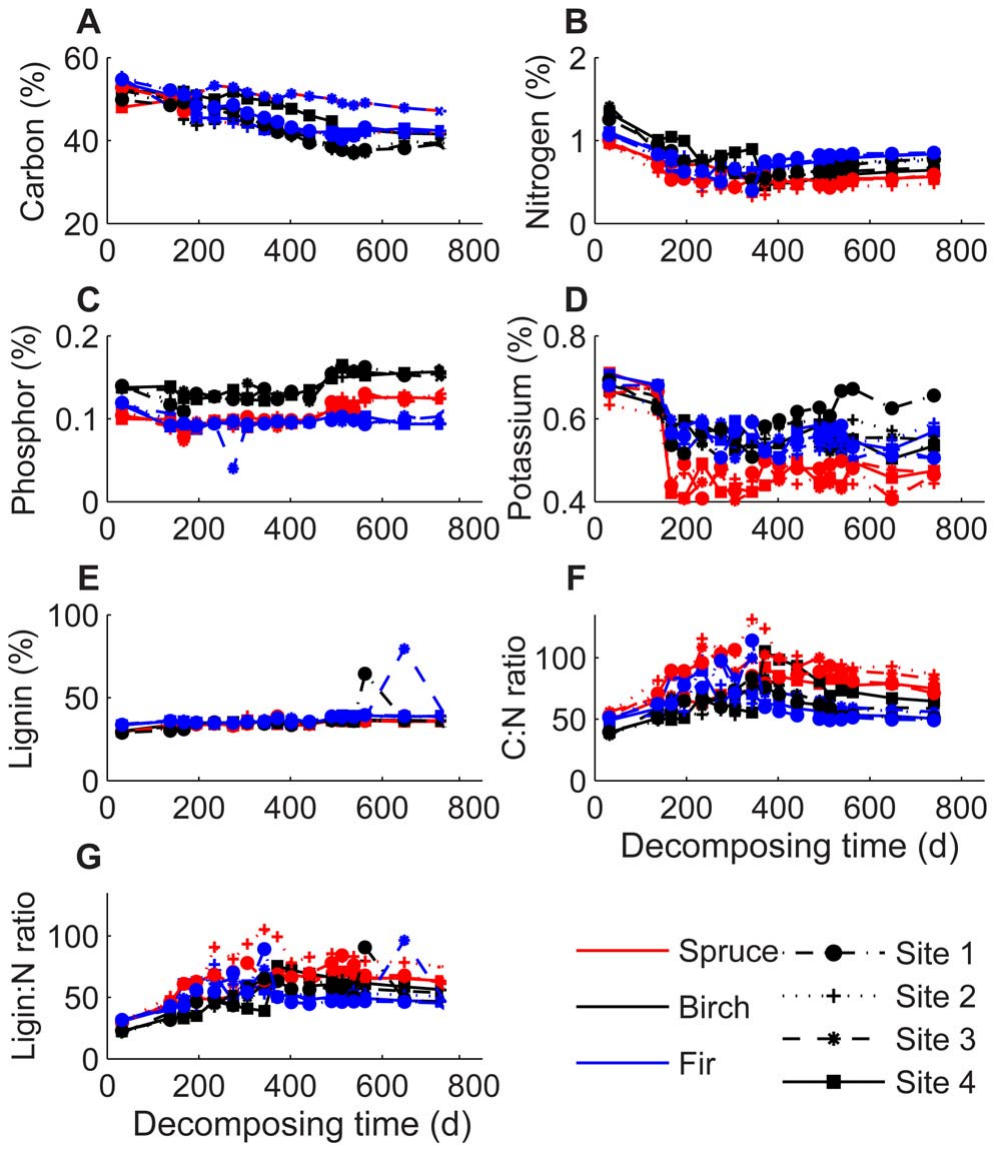
Concentrations of leaf litter carbon (A), nitrogen (B), phosphorus (C), potassium (D), lignin (E), C:N (F) and lignin:N (G) ratios at different elevations after every sampling date.

**Figure 4:**
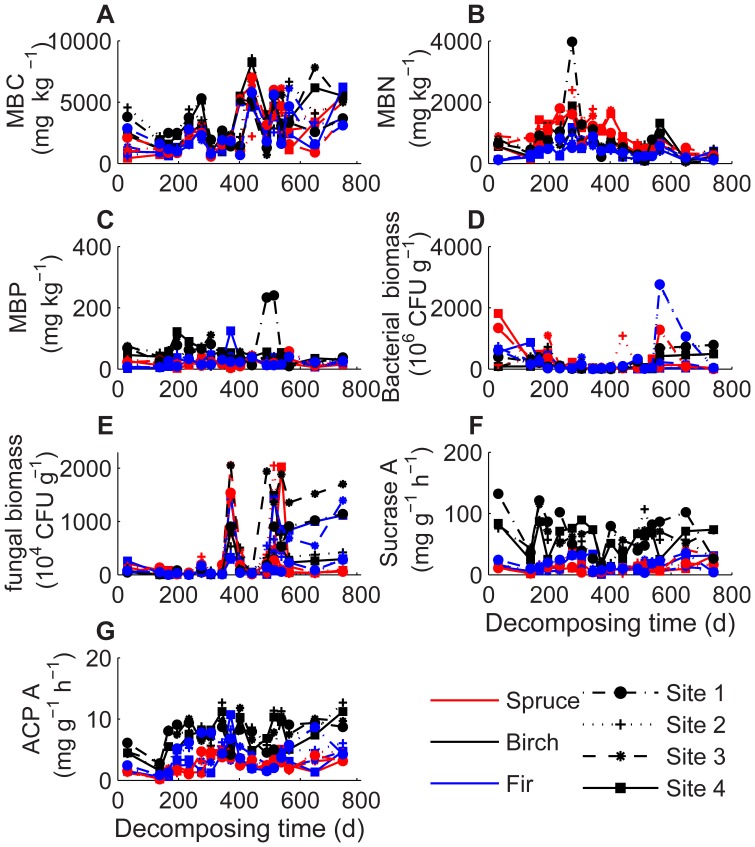
Concentrations of leaf litter microbial biology, activity of sucrase and acid phosphatase at different elevations after every sampling date. Abbreviations: MBC = microbial biomass carbon (A), MBN = microbial biomass nitrogen (B), MBP = microbial biomass phosphorous (C), bacterial biomass (D), fungal biomass (E) sucrase A = Sucrase activity (F) and ACPA = acid (pH 6.5) phosphatase activity (G).

Changes of C, N, P, K, lignin, microbial biomass, and bacteria and fungi fluctuated over the whole decomposition time (Figure 3 and 4). Regardless of the elevation, *k* value was significantly positively affected by soluble N, P and K concentrations during the winter decomposition, but significantly negatively affected by lignin, lignin:N and C:N ratios during the first winter and first growing season (*P*<0.05, Table 4). However, the Pearson’s correlation coefficients explained only a small portion of variance or almost no statistical correlations between the biomass loss rate and all decomposing litter chemical variables during the second year of decomposition (Table 4). Analysis of Pearson’s correlation coefficients explained a portion of variance in the decomposition rate. In general MBC, MBN and MBP in the four elevations had significantly positive effects on the litter decomposition rate during the 2-year decomposition (*P*<0.05, Table 4). Activity of sucrase and ACP in all four elevations also had positive effects on the decomposition rate during the first growing season of decomposition (*P*<0.05, Table 4).

**Figure 5:**
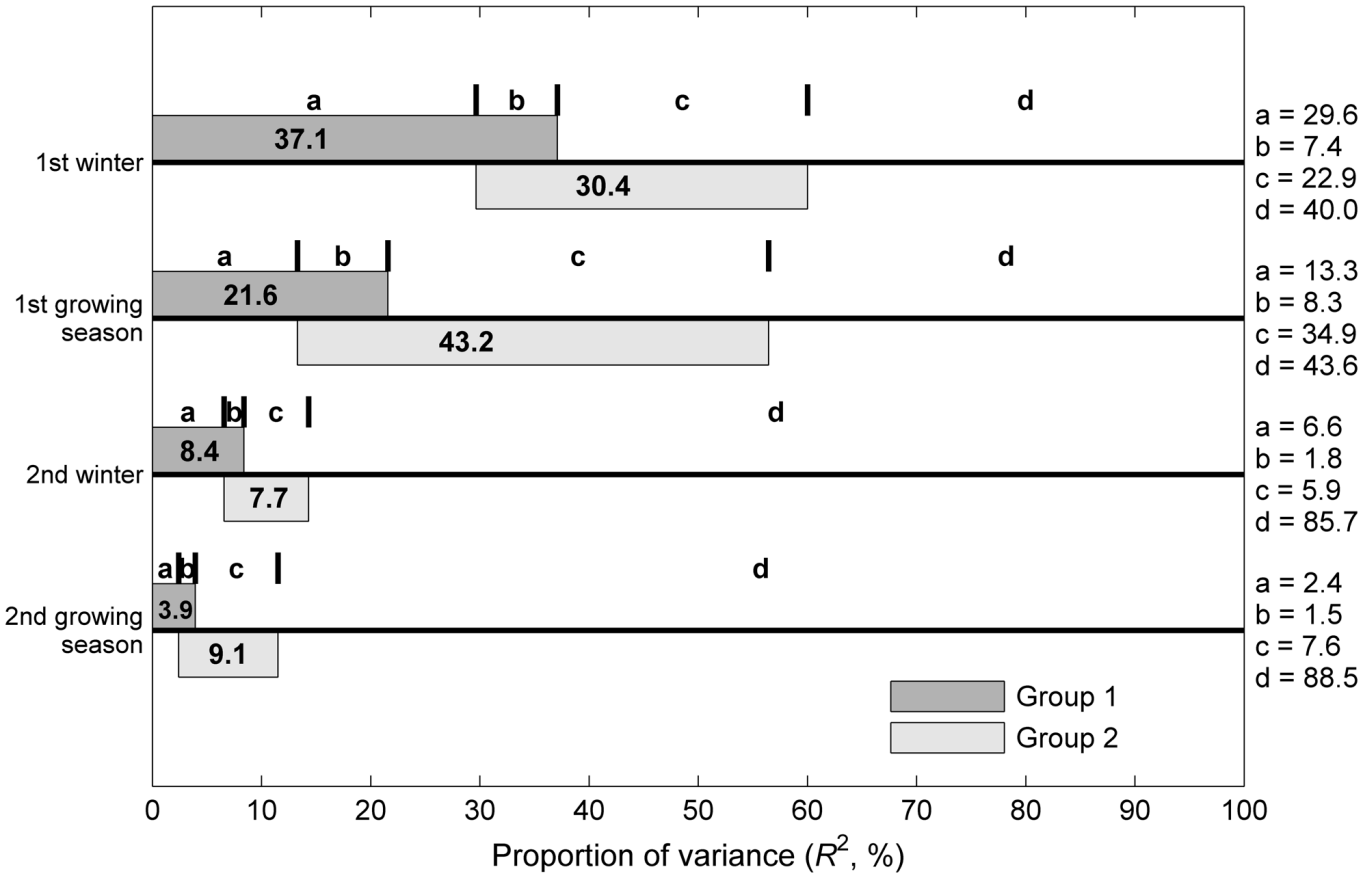
Partitioning of deviance in the decomposition rate during each stage calculated with a partial regression method. In the figure, a and c are the independent components attributed to two groups of biotic factors (litter chemistry and microbe-related factors), respectively; b is the covariance in a component of the two groups; and d is the residual deviance. The group 1 consists of litter chemical variables, whilst the group 2 is the microbe-related factors. See details of these partial regressions in the Material and Methods section.

The relative effects of litter chemistry and microbe-related factors on the decomposition rate (*k*) during each decomposition stage were shown in Figure 5. Effects of the biotic factors were divided into two groups. The group 1 (litter chemistry) consisted of litter chemical elements, whilst the group 2 included litter microbial biomass, extracellular enzyme activity and decomposer compositions. First, when the effects of the group 2 were under control, the group 1 independently explained 29.6% (the first winter season) and 13.3% of the explained deviance (the first growing season) (Fraction a). In contrast, less than 10% of the explained deviance was detected during the second year of decomposition. By comparison, when the effects of the group 1 were under control, 22.9% and 34.9% of the explained deviances were attributed to the group 2 during the first winter and the first growing season, respectively (Fraction c). Likewise, only 5.9% (the winter) and 7.6% (the growing season) of the explained deviance were attributed to the group 2 during the second year of decomposition. In total, biotic factors explained 60.0% of the variation in the decomposition rate during the first winter and 56.4% during the first growing season (100% – Fraction d, R^2^(III)), while the R^2^ of the explained deviance decreased with time of decomposition (<20% during the second year, R^2^(III)).

## Discussion

Our results supported the first hypothesis that the abiotic factors including soil temperature and soil freeze-thaw cycles (FTCs) influenced litter decomposition rate in these four subalpine forests (Table 4). We further found that the soil FTC was an important abiotic factor during the winter decomposition, while the mean soil temperature controlled the litter decomposition during the growing season (Table 4). Over winters, the labile component of litter, cold-tolerance microorganisms [36], frequent freeze-thaw cycles [37] or physical leaching events [24] lead to a rapid litter decomposition. The improvement of litter decomposability by soil FTCs is also supported by our previous observations [22], [38] and other studies [23], [39], [40] at high altitude and latitude regions. Significantly positive correlations between the numbers of FTCs and decomposition rate (*k*) indicated that physical processes associated with the freeze-thaw cycling seemed to be one of the most important abiotic factors affecting litter decomposition in the winter season under these subalpine forests. Significantly positive effect of soil temperature on *k* value was detected during the growing seasons, but not during the winter season (Table 4). Patterns of litter decomposition between the winter season and the growing season could be thus established with soil freeze-thaw patterns during winter [2], [37] and soil temperature during the growing season [41] in these subalpine forests.

As for the second hypothesis, we demonstrated that the correlations between *k* value and a number of abiotic and biotic factors affecting the change of litter quality. These factors include chemical structure elements of leaf biomass, microbial biomass, extracellular enzyme activity and decomposer compositions in litter. Studies have identified the initial litter chemistry (mainly including lignin concentration, ratios of C:N and lignin:N) as the most reliable predictors of decomposition rates [42]–[44]. Litter chemical characteristics are of great importance in controlling both the short- and long-term decomposition rates [43], [45], and plant species traits controlled the decomposition rate by correlating with different species ecological strategies [46]. On the other hand, decomposer activity beneath snow, rather than freeze-thaw cycles, might be a more important factor for the wintertime decomposition [47]. Although we did not measure the snowpack properties along with the elevation gradient in this study, litter decomposition related to microbial factors were explored for testing the correlations between decomposition in the winter and subsequent growing season and decomposer activities. In general, MBC was positively related to elevation and positively affected the decomposition rate during the first winter of decomposition (Table 3, 4), indicating an effect of snowpack on the microbial composition during winter [47], [48]. It seemed that both litter qualities and microbe-related factors in litter were affected by the elevation gradient, and then the changed biotic factors in litter controlled the subsequent decomposition process.

In addition, the results of the multiple regression analyses indicated the temporal dynamics of biotic factors on decomposition in this subalpine forest (Figure 5). The patterns between the winter and the subsequent growing season differed in this subalpine forest. Bray et al. [13] used a nonmetric multidimensional scaling (NMS) method, which decreases litter chemistry and microbe-related variables to a single axis, to examine the relationships between microbes, litter chemistry, and decomposition rates, and found that the temporal dynamics of litter chemistry and microbial communities on *k* differed between the early and later of decomposition. The early decomposition (two months) was determined by the initial litter chemistry and the later decomposition (eight months) was controlled by the microbial community [13]. Therefore, our study indicated that the litter decomposition experienced two different decomposition stages in these subalpine forests. Litter decomposition in this subalpine forest can thus be described by a two stage mechanism that switches from the winter of decay to the later growing season after a majority of the labile material has been decomposed by the freezing and thawing events during the winter [23]. It is apparent that both the decomposition freeze-thaw event [28], [49], [50] and litter chemistry [13] play important roles in controlling decomposition and determining the decomposer activity beneath snowpack in the winter season [51]–[53], which then exerts a further role in determining the decomposition rate in the growing season.

However, further studies are warranted: 1) Although “home field advantage” on litter decomposition had been reported by previous studies [54]–[56], the experimental design of this study did not allow us to test this hypothesis. The selected species in this experiment dominate in each elevation, except for the birch and spruce in site 1 (3,582 m elevation) and low proportion of the fir in site 4 (2,704 m elevation). Although plant species might promote certain microbial communities and conditions that favor decomposition of their litter, microbe-related factors had been considered during each stage in this study. 2) Due to lack of a controlled experiment without the FTCs to test the hypothesis that freezing and thawing promotes decomposition in this study, it is hard to separate the effects of freeze-thaw cycles from the effects of warmer periods when microbial growth would be more rapidly. Nevertheless, studies have found that soil FTCs could improve litter decomposition in the laboratory experiments [37]. At present limited information is available about the effects of soil FTCs and soil temperature on litter decomposition during winter in cold biomes. Future studies should employ controlled experiments concerning with FTCs and ‘native species’ litter in order to test the effects of soil FTCs on and to predict litter decomposition more accurately in subalpine forests.

### Conclusions

Litter decomposition in subalpine forests experienced annually two different decomposition stages, e.g. the winter and the subsequent growing season. Frequent freeze-thaw cycles and decomposing litter chemistry play important roles in determining decomposition during the winter season. Relative higher soil temperature and precipitation within an elevation gradient may not increase the decomposition rate in the subalpine forest during the winter, unless accompanied by an increase of soil freezing and thawing cycles. This study also suggests that frequent freeze-thaw cycles changed litter chemical and microbial compositions, which, in turn, determine the decomposition rate once labile energy and nutritional sources are exhausted in the winter and the subsequent growing season. The microbial activity and composition explains a large portion of variations in litter decomposition throughout the two decomposition stages especially during the growing season. Our results may have important implications for addressing biogeochemical nutrient cycling in highland cold ecosystems under global warming scenarios.
